# Effects of playing experience on joint kinetics and ball-release velocity in mid- and long-range basketball jump shots

**DOI:** 10.7717/peerj.20757

**Published:** 2026-02-05

**Authors:** Pengzhou Chen, Tao Chen, Xuan Tang, Ming Li, Xiangjun Miao

**Affiliations:** 1 Beijing Sport University, Beijing, China; 2 College of Education and Sports Sciences, Yangtze University, Jingzhou, Hubei, China; 3 School of Physical Education, Yunnan University, Kunming, China

**Keywords:** Rate of torque development, Peak power, Angular impulse, Release velocity

## Abstract

**Purpose:**

This study investigated how playing experience influences joint kinetics and ball-release velocity during mid- and long-range jump shots. Wrist, elbow, shoulder and knee rate of torque development (RTD), peak power (P_peak_), and angular impulse (AI) were quantified, along with vertical release velocity (VV) and horizontal release velocity (HV) at release.

**Methods:**

In a cross-sectional design, 15 experienced and 15 novice male collegiate basketball players each performed three made jump shots from 4.8 m and 6.75 m. A 3-D motion-capture system synchronised with force plates provided the data used to compute RTD, P_peak_, AI, VV and HV. Outcomes were compared with a two-way mixed ANOVA.

**Results:**

Experienced players exhibited greater wrist AI (*p* < 0.001), elbow RTD (*p* = 0.002), P_peak_ (*p* = 0.045) and AI (*p* < 0.001), knee P_peak_ (*p* = 0.002) and VV (*p* < 0.001). Longer shooting distance increased shoulder P_peak_ (*p* = 0.036) and HV (*p* = 0.018).

**Conclusions:**

Collectively, these results show that experience enhances joint kinetic output, providing the mechanical foundation for more efficient and dependable shooting. For novice players, emphasising wrist-endurance work, explosive-power training for the elbow and knee, and targeted drills to raise VV is recommended to improve overall on-court shooting performance.

## Introduction

In basketball games, teams accumulate points by shooting the ball into the basket, making shooting a key skill that directly influences the outcome of the game (Okazaki & Rodacki, 2012; Okazaki, Rodacki & Satern, 2015). Among the available techniques, the jump shot couples exceptional scoring efficiency with broad tactical versatility, making it the single most frequent and influential scoring action in competition (Xv et al., 2025). Indeed, analytical data show that almost half of all points in a game arise from jump shots, highlighting their indispensable strategic value (Tang & Shung, 2005; Erčulj & Štrumbelj, 2013). Mastery of the jump shot is therefore central to on-court success, irrespective of playing position.

In actual gameplay, players must execute jump shots from various distances to spread the defense and complete tactical plays. As this situational constraint (shooting distance) changes, players flexibly adjust their movement patterns (Okazaki, Rodacki & Satern, 2015; Xv et al., 2025), manifesting as higher shoulder angular velocity (Okazaki, Rodacki & Satern, 2015), enlarged shoulder and wrist ranges of motion (Cabarkapa et al., 2022), and greater elbow extension (Caseiro et al., 2023). These adaptations reflect compensatory modulation by the nervous system under intensified task constraints (Okazaki & Rodacki, 2012). In match play, approximately 4.8 m and 6.75 m represent typical mid- and long-range jump-shot situations and are used frequently (Nakano, Fukashiro & Yoshioka, 2020; França et al., 2022). Previous studies have revealed differences in muscle-synergy patterns and contraction performance at these two distances (Xv et al., 2025; Li et al., 2025); however, research on the joint-level kinetics remains relatively scarce, and clarifying this is directly relevant to enhancing competitive performance.

Shooting performance exhibits high inter-individual variability; different players may adopt different technical patterns yet still achieve similar shooting accuracy (Okazaki, Rodacki & Satern, 2015). Playing experience is one of the key factors contributing to technical differences and has a significant impact on jump shot performance. Novices often struggle to reach peak joint angular velocity at the moment of ball release, which limits their release speed and height (Okazaki & Rodacki, 2005); in contrast, experienced players demonstrate greater movement consistency and repeatability (Slegers, Lee & Wong, 2021; Marineau et al., 2024). Neuromotor evidence shows that beginners simplify control by “freezing” joint degrees of freedom (Vereijken et al., 1992; Newell & Vaillancourt, 2001) and by diminishing shoulder–elbow–wrist coupling during the release phase (Okazaki & Rodacki, 2005). This control scheme curbs the deployment of efficient shooting mechanics and slows the transfer of high-level skills (Okazaki, Rodacki & Satern, 2015). Although previous studies have revealed that experienced players boost release speed on long-range shots by refining movement patterns (Caseiro et al., 2023) and that novices reconfigure muscle synergies to compensate (Xv et al., 2025), such work has focused mainly on kinematic and coordination aspects. Little is known about how joint kinetics change with distance and experience to govern release speed. Because intrinsic kinetics are indispensable for generating velocity and controlling accuracy (Okazaki, Rodacki & Satern, 2015), a deeper understanding of how playing experience shapes joint kinetics and release speed in mid- and long-range shooting is essential for upgrading novice performance.

This study investigates the influence of playing experience on jump-shot biomechanics. We quantified wrist, elbow, shoulder, and knee rate of torque development (RTD), peak power (P_peak_), and angular impulse (AI) during mid- and long-range shots, along with the ball’s vertical release velocity (VV) and horizontal release velocity (HV). We hypothesised that experienced players would attain higher VV and that both VV and HV would increase as shooting distance lengthens, with these velocity gains tightly coupled to joint-level kinetics.

## Materials & Methods

### Participants

Thirty right-hand–dominant male collegiate basketball players participated. To sharpen the experience contrast, we grouped them by verified match exposure in the Chinese National Collegiate League (CUBA). The experienced group (EG; *n* = 15; 22.3 ± 1.6 years; 185.8 ± 4.3 cm; 78.7 ± 5.7 kg) were first-team roster members who had completed at least one full CUBA season and had ≥ 5 years of continuous organised play. The novice group (NG; *n* = 15; 19.2 ± 0.6 years; 183.4 ± 2.6 cm; 76.5 ± 5.1 kg) comprised newly selected squad players preparing for their debut CUBA season; they had no prior official match experience but had demonstrated competent basic jump-shot technique during familiarisation. An a priori power analysis (G*Power 3.1.9.7; *α* = 0.05; 1–*β* = 0.80; *f* = 0.30, derived from pilot data) determined that a minimum of 28 participants was necessary; 30 were recruited to maximise power and mitigate potential dropout. All players were full-time students engaged in organised basketball training four to five times per week (2.5–3.5 h per session) and had no history of neuromuscular disorders. After receiving a detailed explanation of the procedures, each participant provided written informed consent.

Participants were recruited between May 15 and June 15, 2025, and all data collection was conducted between June 16 and July 15, 2025 at the Motion Analysis Laboratory, Yunnan University. The study conformed to the Declaration of Helsinki and was approved by the Institutional Review Board of Yunnan University (Approval No.: 2024271).

### Testing setup

**Figure 1:**
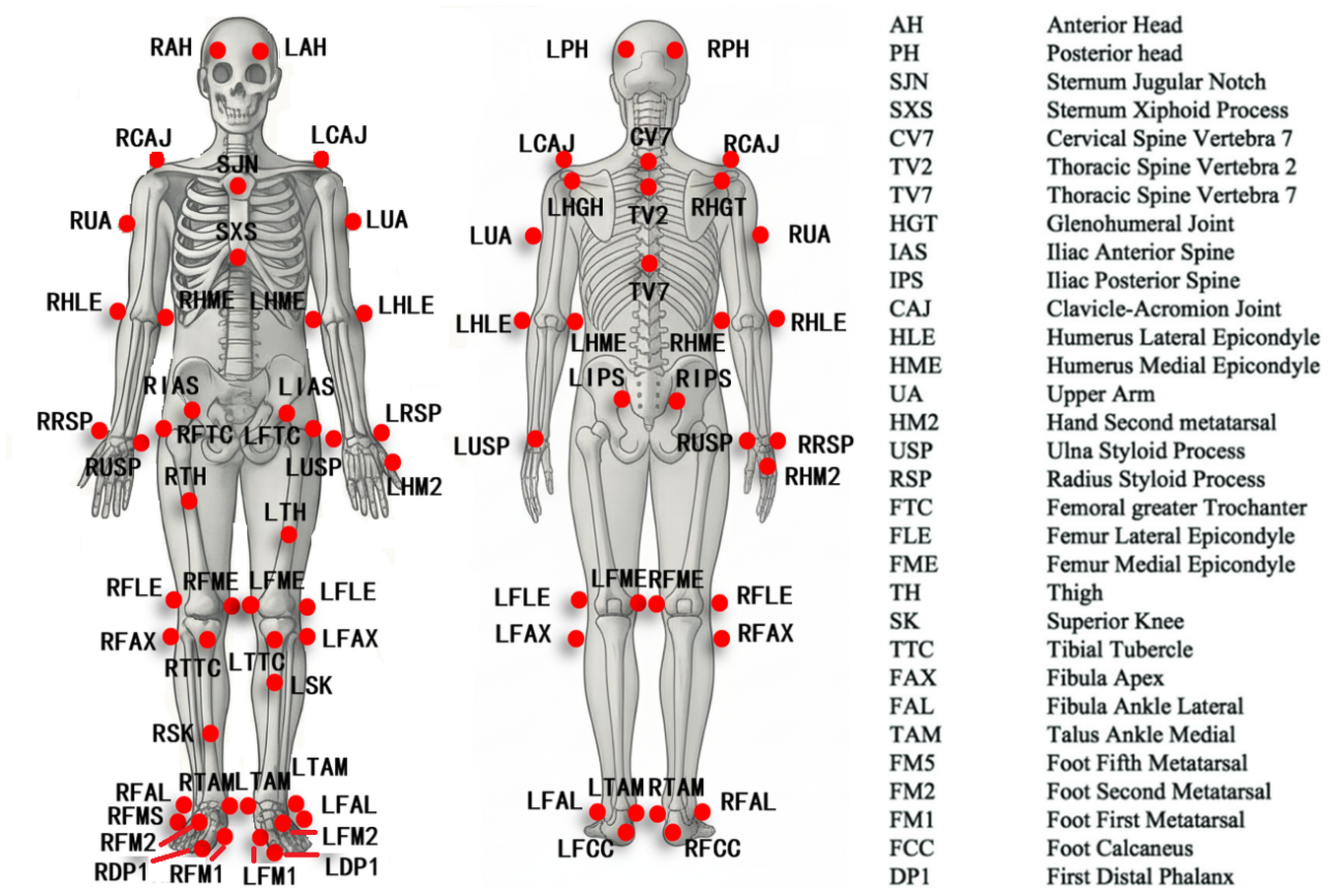
Anatomical position of the reflex marker (*N* = 57). “L” and “R” represent the left and right sides.

Jump-shot biomechanics were captured with an integrated three-dimensional motion-analysis system. Thirteen OptiTrack cameras (NaturalPoint, Inc., Corvallis, OR, USA) sampling at 240 Hz tracked fifty-seven 14 mm markers attached to key anatomical landmarks illustrated in Fig. 1. OptiTrack is widely regarded as a gold-standard motion-capture system for dynamic sports movements and has demonstrated sub-millimetre spatial accuracy (mean absolute error ≲ 0.6 mm) (Piche et al., 2022). Before each session, the camera array was calibrated with a dynamic wand and a static L-frame; the global coordinate system and ground plane were then defined, and both force plates were zeroed. Two force plates (AMTI Inc., Newton, MD, USA) simultaneously recorded ground reaction force at 2,400 Hz; the plates are factory-calibrated and maintain linearity error below 1% across the measurement range, with inter-device latency kept under 6 ms. All kinematic and kinetic data streams were synchronised in Motive 2.2.0 (NaturalPoint, Inc., Corvallis, OR, USA) to ensure precise temporal alignment for subsequent analyses.

### Testing protocol

The experiment was conducted in an indoor, climate-controlled biomechanics laboratory using a regulation-height basket (3.05 m) and standard men’s size-7 basketballs. The mid- and long-range shooting lines (4.8 m and 6.75 m) were measured relative to the force-plate position and marked on the floor to guide foot placement. The force plates were embedded flush with the surrounding surface to avoid step artefacts and to better approximate on-court foot contacts. Prior to data acquisition, all participants received a briefing and live demonstration of the experimental workflow. After confirming understanding, they completed a 5-min dynamic warm-up on a treadmill, with speed and incline tailored to individual comfort, followed by a 5-min passive rest to restore baseline status. Players then executed up to five familiarisation jump shots from both mid- and long-range to acclimate to the rim and environment, followed by a 3-min rest. During formal testing, each participant was randomly assigned the starting shooting distance (mid- or long-range). Standing on the force plates, they performed a stationary, game-like jump shot: a two-foot take-off, the ball released above the head with full elbow extension and wrist flexion, and a natural landing back on the plates. Each attempt began with a shoulder-height chest pass from a trained assistant positioned 3 m in front of the shooter; no dribble was allowed, and the same assistant delivered all passes to keep timing and trajectory consistent. Each distance required three successful jump shots (made basket). To preserve data integrity, trials at the same distance were separated by 1 min of passive rest, and blocks at different distances were separated by 3 min.

### Data processing

Kinetic and ball-release variables were processed in Visual3D Setup x64 v2023.9.1 (C-Motion, Germantown, MD, USA). Joint moments and power were obtained *via* inverse dynamics, whereas ball speed was extracted from marker trajectories; the velocity of a rigid-body model of the ball at release was decomposed into VV and HV. Analysis windows were set as follows: (i) knee, from the onset of countermovement to the instant ground-reaction force fell below 20 N (Zhang et al., 2025); (ii) shoulder, elbow and wrist, from the flexion-to-extension switch in the standing phase to ball release. Signals were zero-phase filtered with a fourth-order Butterworth filter (cut-off 17 Hz for moment- and power-time series; 6 Hz for velocity-time series, including ball speed) (Li et al., 2024). Reliability of release-speed metrics was examined across three valid shots per condition using ICC(3,3) and CV (CV % = SD/mean ×100). If ICC ≥ 0.80 and CV ≤ 10%, the three trials were averaged; otherwise, the participant returned within 48 h, at the same time of day, to perform one additional (fourth) trial for the affected distance. If the recalculated reliability still fell below threshold, that metric was flagged as missing and excluded. RTD and AI were derived from moment-time curves, and P_peak_ from power-time curves. RTD was calculated as the change in joint moment from onset to its peak, divided by the elapsed time between those two instants (ΔTorque/ΔTime). These variables were averaged across trials and normalised to body mass to facilitate group comparison. All computations were completed in Microsoft Excel 2019 (Microsoft, Redmond, WA, USA).

### Statistical analysis

All results are presented as mean ±SD. Analyses were conducted in R 4.3.2 (R Core Team, 2023). Normality of each variable (RTD, P_peak_, AT, VV, HV) was checked with the Shapiro–Wilk test, with *p* > 0.05 indicating an approximately normal distribution. A two-way mixed-model ANOVA evaluated the main and interactive effects of playing experience (novice *vs.* experienced) and shooting distance (mid- *vs.* long-range). Significant interactions (*p* < 0.05) were followed by simple-effects tests, and Bonferroni-corrected pairwise comparisons. Statistical significance was accepted at *p* < 0.05. Effect sizes were reported as partial eta-squared (*η*_p_^2^), reflecting the variance in the dependent variables explained by each factor, and interpreted using conventional thresholds as small (≈0.01), medium (≈0.06), and large (≈0.14) (Richardson, 2011).

## Results

Table 1 reports the mean ±SD values for wrist RTD, P_peak_ and AI. For RTD, the main effect of group was significant (*p* = 0.047), with a very large effect size (*η*_p_^2^ = 0.54), indicating that NG was significantly greater than EG. For AI, the main effect of group was also highly significant (*p* < 0.001), accompanied by a very large effect size (*η*_p_^2^ = 0.54), showing that EG was significantly greater than NG. For P_peak_, although the main effect of group was not significant (*p* > 0.05), it still demonstrated a medium effect size (*η*_p_^2^ = 0.07). In contrast, the main effect of shooting distance was not significant for any wrist variable (*p* > 0.05), and the effect size was small in all cases (*η*_p_^2^ = 0.02), suggesting no practically meaningful change in wrist explosive strategy from mid- to long-range.

**Table 1:** Wrist joint explosive performance in jump shots: comparing two distances and groups (mean ± SD).

		Jump Shot Distance (m)		
Parameter	Group	4.8	6.75	*η*_p_^2^
RTD (N m kg^−1^ s^−1^)	NG	0.58 ± 0.14	0.76 ± 0.43	0.54 (Group)
	EG	0.53 ± 0.23^*^	0.55 ± 0.19^*^	0.02 (Distance)
P_peak_ (W kg^−1^)	NG	0.22 ± 0.09	0.37 ± 0.34	0.07 (Group)
	EG	0.35 ± 0.15	0.42 ± 0.19	0.02 (Distance)
AI (N s kg^−1^)	NG	0.0004 ± 0.0002	0.0007 ± 0.0006	0.54 (Group)
	EG	0.0015 ± 0.0004^*^	0.0017 ± 0.0006^*^	0.02 (Distance)

**Notes.**

RTD Rate of torque development P_peak_ Peak power AI Angular impulse *η*_p_^2^ Partial eta squared NG Novice Group EG Experienced Group

* A significantly (*p* < 0.05) different from the NG.

Table 2 summarises elbow RTD, P_peak_ and AI. All three variables displayed significant group effects, with higher values in EG than NG (RTD: *p* = 0.002; P_peak_: *p* = 0.045; AI: *p* < 0.001). These group effects were also practically meaningful, with effect sizes ranging from medium (P_peak_: *η*_p_^2^ = 0.09) to large (RTD: *η*_p_^2^ = 0.12; AI: *η*_p_^2^ = 0.18). In contrast, neither distance (RTD: *p* = 0.681; P_peak_: *p* = 0.231; AI: *p* = 0.399) nor the interaction terms (RTD: *p* = 0.910; P_peak_: *p* = 0.419; AI: *p* = 0.699) were significant. Furthermore, the effect size for distance was small for all three variables (RTD: *η*_p_^2^ = 0.02; P_peak_: *η*_p_^2^ = 0.02; AI: *η*_p_^2^ = 0.04), indicating no practically meaningful change in elbow explosive performance from mid- to long-range.

**Table 2:** Elbow joint explosive performance in jump shots: comparing two distances and groups (mean ± SD).

		Jump Shot Distance (m)		
Parameter	Group	4.8	6.75	*η*_p_^2^
RTD (N m kg^−1^ s^−1^)	NG	1.09 ± 0.49	1.19 ± 0.37	0.12 (Group)
	EG	1.41 ± 0.31^*^	1.54 ± 0.37^*^	0.02 (Distance)
P_peak_ (W kg^−1^)	NG	0.92 ± 0.4	0.99 ± 0.29	0.09 (Group)
	EG	1.02 ± 0.28^*^	1.25 ± 0.48^*^	0.02 (Distance)
AI (N s kg^−1^)	NG	0.0051 ± 0.0014	0.0055 ± 0.0017	0.18 (Group)
	EG	0.0067 ± 0.0018^*^	0.0074 ± 0.0021^*^	0.04 (Distance)

**Notes.**

RTD Rate of torque development P_peak_ Peak power AI Angular impulse *η*_p_^2^ Partial eta squared NG Novice Group EG Experienced Group

* A significantly (*p* < 0.05) different from the NG.

Table 3 presents shoulder RTD, P_peak_ and AI. RTD showed a significant group effect (*p* = 0.014), with a medium effect size (*η*_p_^2^ = 0.09), as NG exceeded EG. The main effect of distance was not significant (*p* = 0.282), though it also demonstrated a medium effect size (*η*_p_^2^ = 0.07). For P_peak_, a significant distance effect emerged (*p* = 0.036), with a medium effect size (*η*_p_^2^ = 0.1), as long-range shots produced greater values than mid-range. The main effect of group (*p* = 0.224) was not significant, and its effect size was small (*η*_p_^2^ = 0.04). For AI, there were no significant main effects; the group effect was trivial (*η*_p_^2^ = 0.00, *p* = 0.967), while the distance effect was medium (*η*_p_^2^ = 0.07) despite not reaching significance (*p* = 0.23). No interaction effects were significant for any variable (all *p* > 0.05).

**Table 3:** Shoulder joint explosive performance in jump shots: comparing two distances and groups (mean ± SD).

		Jump Shot Distance (m)		
Parameter	Group	4.8	6.75	*η*_p_^2^
RTD (N m kg^−1^ s^−1^)	NG	3.12 ± 1.49	4.02 ± 2.22	0.09 (Group)
	EG	2.02 ± 1.16^*^	3.03 ± 1.21^*^	0.07 (Distance)
P_peak_ (W kg^−1^)	NG	0.73 ± 0.39	1.11 ± 0.54^†^	0.04 (Group)
	EG	0.93 ± 0.37	1.26 ± 0.51^†^	0.1 (Distance)
AI (N s kg^−1^)	NG	0.0104 ± 0.0043	0.0133 ± 0.0051	0.00 (Group)
	EG	0.0104 ± 0.0041	0.0135 ± 0.005	0.07 (Distance)

**Notes.**

RTD Rate of torque development P_peak_ Peak power AI Angular impulse *η*_p_^2^ Partial eta squared NG Novice Group EG Experienced Group

* A significantly (*p* < 0.05) different from the NG.

† A significantly (*p* < 0.05) different from the 4.8 m.

Table 4 shows the mean ±SD of knee RTD, P_peak_ and AI. Neither RTD nor AI was significantly influenced by playing experience or shooting distance, and no interaction effects emerged (all *p* > 0.48). The associated effect sizes for these non-significant factors were also small to trivial (Group RTD: *η*_p_^2^ = 0.04; Group AI: *η*_p_^2^ = 0.00; Distance RTD: *η*_p_^2^ = 0.00; Distance AI: *η*_p_^2^ = 0.02). In contrast, P_peak_ displayed a clear group effect (*p* = 0.002), with experienced players generating higher P_peak_ than novices. This effect was also practically meaningful, demonstrating a medium-to-large effect size (*η*_p_^2^ = 0.13). The effects of distance (*p* ≥ 0.956) and the interaction term were non-significant, and the distance effect size was small (*η*_p_^2^ = 0.01).

**Table 4:** Knee joint explosive performance in jump shots: comparing two distances and groups (mean ± SD).

		Jump Shot Distance (m)		
Parameter	Group	4.8	6.75	*η*_p_^2^
RTD (N m kg^−1^ s^−1^)	NG	8.9 ± 2.03	10.01 ± 2.79	0.04 (Group)
	EG	9.98 ± 2.5	10.24 ± 1.79	0.00 (Distance)
P_peak_ (W kg^−1^)	NG	7.27 ± 1.69	7.89 ± 1.94	0.13 (Group)
	EG	9.08 ± 2.63^*^	9.64 ± 2.16^*^	0.01 (Distance)
AI (N s kg^−1^)	NG	0.17 ± 0.06	0.18 ± 0.06	0.00 (Group)
	EG	0.16 ± 0.05	0.17 ± 0.05	0.02 (Distance)

**Notes.**

RTD Rate of torque development P_peak_ Peak power AI Angular impulse *η*_p_^2^ Partial eta squared NG Novice Group EG Experienced Group

* A significantly (*p* < 0.05) different from the NG.

**Figure 2:**
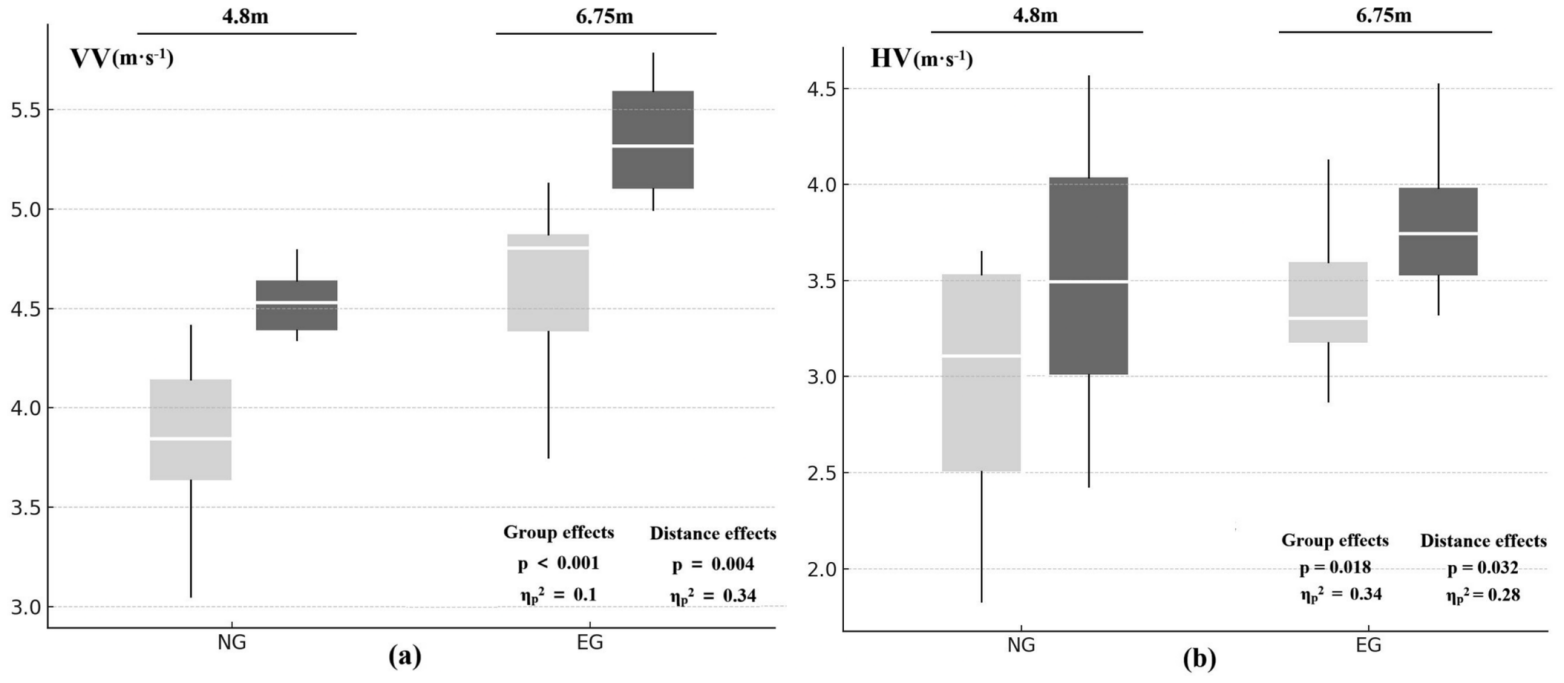
Vertical release velocity (VV) and horizontal release velocity (HV) for two groups during (A) mid- (4.8 m) and (B) long-distance (6.75 m) jump shots. Each box plot illustrates the data distribution, where the upper and lower edges represent the upper (Q3) and lower (Q1) quartiles, respectively, and the central white line indicates the median. The whiskers extend to the maximum and minimum values within 1.5 times the interquartile range (IQR). Statistical results for group and distance effects, including *p*-values and partial eta-squared (*η*_*p*_2), are displayed within each panel.

Table 5 presents the ball-release velocities. Expanding the shooting distance to 6.75 m increased VV by 13.62% and HV by 18.24% in novices, while experienced players recorded gains of 16.34% for VV and 11.50% for HV. The two-way ANOVA (Fig. 2) corroborated these patterns, revealing significant main effects of group (VV: *p* < 0.001, *η*_p_^2^ = 0.1; HV: *p* = 0.018, *η*_p_^2^ = 0.34) and distance (VV: *p* = 0.004, *η*_p_^2^ = 0.34; HV: *p* = 0.032, *η*_p_^2^ = 0.28) for both velocity components. These results indicate that both group and distance had a practically meaningful impact, with effect sizes ranging from medium (for Group on VV) to very large (for all other main effects). No significant Group × Distance interactions were observed (VV: *p* = 0.317; HV: *p* = 0.543).

**Table 5:** Vertical and horizontal release velocities in jump shots: comparing two distances and groups (mean ± SD).

		Jump Shot Distance (m)		
Parameter	Group	4.8	6.75	*η*_p_^2^
VV (m s^−1^)	NG	3.89 ± 0.37	4.42 ± 0.52^†^	0.1 (Group)
	EG	4.59 ± 0.47^*^	5.34 ± 0.27^*^^†^	0.34 (Distance)
HV (m s^−1^)	NG	2.96 ± 0.6	3.5 ± 0.68^†^	0.34 (Group)
	EG	3.39 ± 0.36^*^	3.78 ± 0.32^*^^†^	0.28 (Distance)

**Notes.**

VV Vertical Velocity HV Horizontal Velocity NG Novice Group EG Experienced Group *η*_p_^2^ Partial eta squared

* A significantly (*p* < 0.05) different from the NG.

† A significantly (*p* < 0.05) different from the 4.75 m.

## Discussion

This study compared the influence of playing experience on joint kinetics and ball-release speed at two prescribed jump-shot distances. Experienced players possessed higher VV, whereas novices had higher HV, implying that experienced players may have a higher shooting tolerance. The reasons for this difference may originate from joint kinetic factors.

The present study shows that EG players produced larger wrist AI, whereas NG achieved higher RTD. This pattern concurs with Dai & Yin (2024), who reported that experienced shooters exhibit greater index- and middle-finger flexion and that release force is transmitted from the wrist, implying a longer wrist torque-time window. Because AI is the time integral of torque and RTD its time derivative, our findings directly support this view. Unlike Dai & Yin’s (2024) mainly kinematic approach, we used inverse dynamics to characterise wrist kinetics. We further propose that the greater AI in experienced shooters accumulates wrist-flexor action to increase ball spin, reduce horizontal velocity, and widen the rim-entry tolerance (Okazaki, Rodacki & Satern, 2015). This interpretation accords with our empirical results and illustrates how playing experience optimises wrist-level kinetics to yield more efficient and precise shooting.

Our results indicate that experienced players surpass novices in elbow RTD, P_peak_ and AI. This aligns with the work of Button et al. (2003), who showed that greater playing experience improves elbow kinematics and identified the elbow as a decisive lever of ball-release speed. Crucially, we analysed kinetic rather than kinematic data, providing a more direct proxy for muscle contraction (Maffiuletti et al., 2016; Li et al., 2023) and, therefore, more actionable insight for training. Because the elbow is both a core contributor to shooting performance (Button et al., 2003; Caseiro et al., 2023) and a defining trait of elite players (Okazaki, Rodacki & Satern, 2015; Cabarkapa et al., 2022), the superior elbow kinetics of the EG are unsurprising and entirely consistent with expectations.

At the shoulder, experienced players produced quicker RTD and, as distance increased, larger P_peak_. This replicates Okazaki & Rodacki (2005), who observed that experienced shooters achieve peak shoulder angular velocity earlier than novices, and is consonant with Vencúrik et al.’s (2021) report of elevated shoulder angular velocity during long-range attempts. Power is the product of force and velocity (Van der Kruk et al., 2018); our kinetic evidence thus reinforces prior kinematic findings while offering a fuller mechanistic account. We suggest that novices restrict shoulder DOF (Okazaki, Rodacki & Satern, 2015) and down-regulate shoulder involvement (Okazaki & Rodacki, 2005) to simplify neural control (Vereijken et al., 1992; Newell & Vaillancourt, 2001). Although this strategy helps them execute the movement, it inevitably curtails both force and speed production (Okazaki, Rodacki & Satern, 2015).

Regarding the knee joint, our data show that experienced players exhibit significantly higher P_peak_. This finding aligns with Delextrat & Cohen (2008), who reported that elite athletes generate greater absolute torque at angular velocities of 1.05 and 3.14 rad/s. Because power is the product of torque and velocity, their results support our observation. Biomechanically, we speculate that higher knee P_peak_ reflects a larger overall lower-limb impulse during the countermovement and push-off phases, which enables a greater jump height and consequently a higher ball release point. A higher release point increases the entry angle and effective rim area, thereby enhancing shot tolerance (Okazaki, Rodacki & Satern, 2015; Cabarkapa et al., 2022). Thus, by boosting knee P_peak_, experienced players refine their take-off mechanics and, ultimately, their scoring efficiency.

Playing experience also increased VV, consistent with Li et al. (2025). This VV advantage stems from the kinetic superiority we observed: experienced players exhibited higher elbow RTD, P_peak_, and AI, together with greater knee P_peak_, supplying the mechanical power needed for steeper vertical launches. Consequently, they achieve a higher release point and a more favorable entry angle (Cabarkapa et al., 2022; Botsi et al., 2024). Novices, lacking comparable joint power and vertical displacement, tend to boost HV to compensate for distance limitations (Li et al., 2025). However, this increase in HV produces greater horizontal displacement during flight, which flattens the entry angle and reduces shot tolerance. Practically, coaches should therefore emphasize drills that develop vertical power and minimize unnecessary lateral movement at release to optimize entry angles and improve shooting consistency.

This study has several limitations. First, all measurements were obtained in a laboratory setting. While this approach secures high data fidelity, it cannot replicate the multifaceted constraints of live competition. Second, players were stratified solely by playing experience; more advanced classification techniques, such as linear discriminant analysis, were not applied, which may have introduced slight bias. Third, our sample comprised only male collegiate players who compete at a sub-elite level; therefore, the present findings cannot be generalised to professional or female basketball populations. Future research should overcome these shortcomings by using cutting-edge instrumentation, collecting data in authentic game environments and extending sampling to professional players. Such efforts will clarify performance differences across the experience spectrum and yield more practical, precise training recommendations for novices.

## Conclusions

This study shows that playing experience is a decisive determinant of jump-shot joint kinetics. Experienced players exhibited significantly greater wrist AI, higher elbow RTD, P_peak_ and AI, and increased knee P_peak_, providing the mechanical foundation for more efficient shooting; long-range shots further demanded elevated shoulder P_peak_. Experience also elevated vertical release velocity: experienced players exploit higher VV to widen shot tolerance, whereas novices depend on additional HV to compensate. Overall, experience optimises joint kinetics, reshapes force-production strategies, and ultimately manifests in the interplay between VV and HV. Based on these findings, we recommend that coaches and practitioners incorporate plyometric medicine-ball throws and box jumps to enhance elbow and knee RTD and P_peak_; implement high-repetition form-shooting drills or resistance-band wrist flexion exercises to build wrist AI; and use vertical-focused, constraint-based training—such as stationary jump shots with same-spot landings or shooting over cones—to promote a more vertical release and reduce reliance on horizontal velocity, thereby improving shooting efficiency.

##  Supplemental Information

10.7717/peerj.20757/supp-1 Supplemental Information 1 All datasets for novice players All recorded variables obtained during the jump shots at both shooting distances. These data were used for the statistical analyses comparing the two distances and the two experience groups.

10.7717/peerj.20757/supp-2 Supplemental Information 2 All datasets for experienced players All recorded variables obtained during the jump shots at both shooting distances. These data were used for the statistical analyses comparing the two distances and the two experience groups.

## Additional Information and Declarations

### Data Availability

The following information was supplied regarding data availability: The raw measurements are available in the Supplementary Files.
